# Supplementary material for: A qualitative analysis of community health worker perspectives on the implementation of the preconception and pregnancy phases of the Bukhali randomised controlled trial
Source: PLOS Glob Public Health. 2024 Mar 14;4(3):e0002578. doi: 10.1371/journal.pgph.0002578 (PMC10939222; doi:10.1371/journal.pgph.0002578)

S1 Fig: Overview of the community health worker approach to the *Bukhali* trial. Source: Draper CE, Thwala N, Slemming W, Lye SJ, Norris SA. Development, implementation, and process evaluation of Bukhali: an intervention from preconception to early childhood. *Glob Implement Res Appl*. 2023. doi:10.1007/s43477-023-00073-8. License: http://creativecommons.org/licenses/by/4.0/. No changes made.

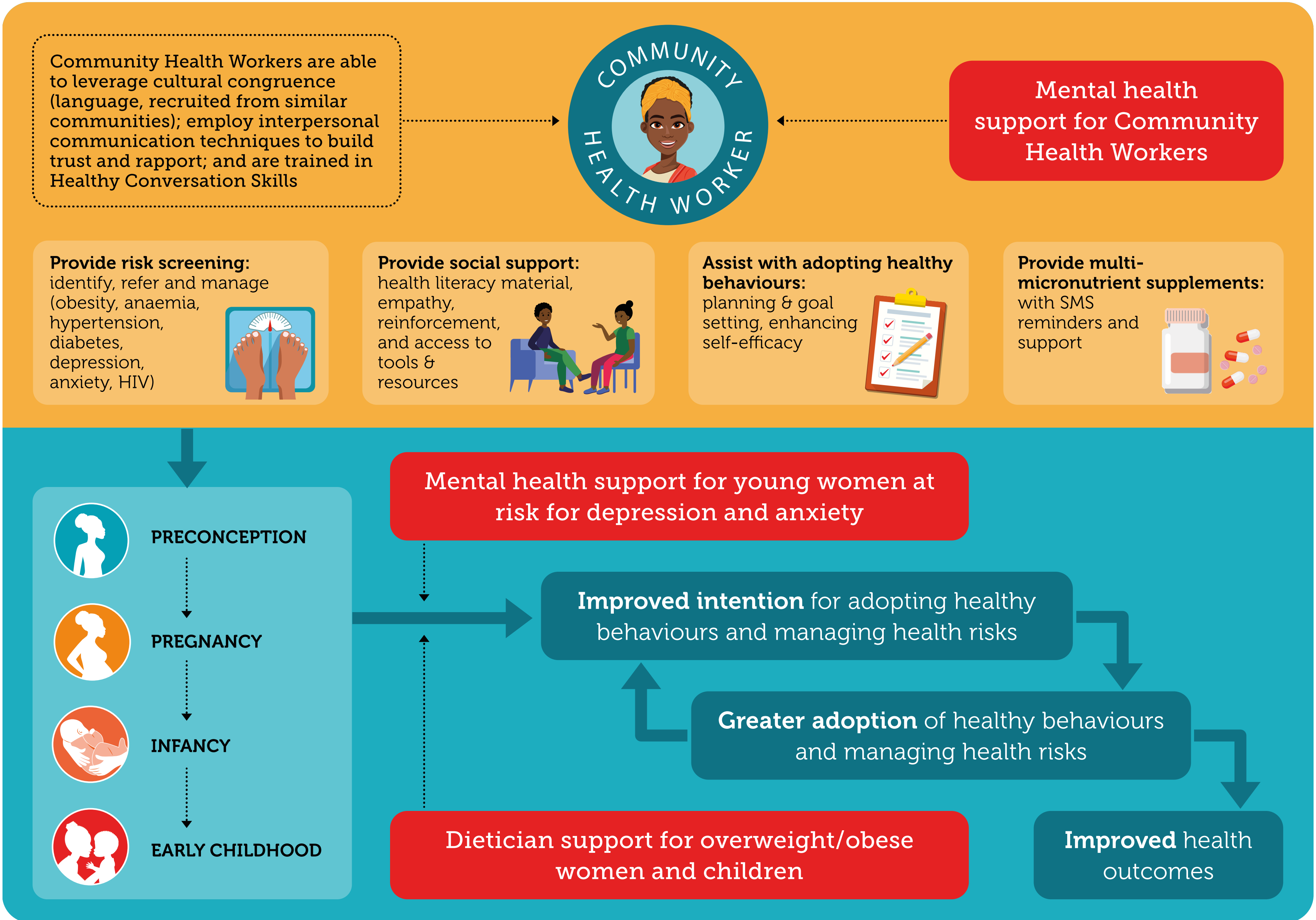

Supplement: S1 Fig — Source: Draper CE, Thwala N, Slemming W, Lye SJ, Norris SA. Development, implementation, and process evaluation of Bukhali: an intervention from preconception to early childhood. Glob Implement Res Appl. 2023. doi:10.1007/s43477-023-00073-8. License: http://creativecommons.org/licenses/by/4.0/. No changes made. (PDF) [file pgph.0002578.s001.pdf]
