# Supplementary material for: A qualitative analysis of community health worker perspectives on the implementation of the preconception and pregnancy phases of the Bukhali randomised controlled trial
Source: PLOS Glob Public Health. 2024 Mar 14;4(3):e0002578. doi: 10.1371/journal.pgph.0002578 (PMC10939222; doi:10.1371/journal.pgph.0002578)
Supplement: S1 Text — (DOCX) [file pgph.0002578.s002.docx]

**Consolidated criteria for reporting qualitative studies (COREQ): 32-item checklist**

Developed from:

Tong A, Sainsbury P, Craig J. Consolidated criteria for reporting qualitative research (COREQ): a 32-item checklist for interviews and focus groups. *International Journal for Quality in Health Care*. 2007. Volume 19, Number 6: pp. 349 – 357

| **No.  Item** | **Criteria description** | **Study Information** |
| --- | --- | --- |
| **Domain 1: Research team and reﬂexivity** |  |  |
| *Personal Characteristics* |  |  |
| 1. Interviewer/ facilitator | Which author/s conducted the interview or focus group? | Larske Soepnel and Khuthala Mabetha |
| 2. Credentials | What were the researcher’s credentials? E.g. PhD, MD | Khuthala Mabetha: PhD  Larske Soepnel: PhD, MD. |
| 3. Occupation | What was their occupation at the time of the study? | Both authors conducting the focus groups were postdoctoral researchers at the time of data collection, mentored by Catherine Draper, who has +20 years of experience in qualitative research |
| 4. Gender | Was the researcher male or female? | Both are female |
| 5. Experience and training | What experience or training did the researcher have? | They have 2-4 years of experiencing conducting qualitative research |
| *Relationship with participants* |  |  |
| 6. Relationship established | Was a relationship established prior to study commencement? | Both authors were known to participants as postdoctoral researchers who have been working on this trial for the past 2 years |
| 7. Participant knowledge of the interviewer | What did the participants know about the researcher? e.g., personal goals, reasons for doing the research | Participants knew that the research team (all co-authors) were interested in their perceptions and experiences of implementing the trial, and that the data collected would be analysed and published in an academic journal |
| 8. Interviewer characteristics | What characteristics were reported about the interviewer/facilitator? e.g., Bias, assumptions, reasons and interests in the research topic | The reasons and interests in the research topic were stated at the beginning of the focus group discussions, and the facilitators’ role in the trial was clarified. |
| **Domain 2: study design** |  |  |
| *Theoretical framework* |  |  |
| 9. Methodological orientation and Theory | What methodological orientation was stated to underpin the study? e.g. grounded theory, discourse analysis, ethnography, phenomenology, content analysis | The methodological orientation of the study was reflexive thematic analysis, additionally drawing on a codebook approach to explore the pre-determined, process-evaluation driven questions. This study was situated within the process evaluation of the *Bukhali* trial, which is informed by the UK MRC’s guidance on process evaluation |
| *Participant selection* |  |  |
| 10. Sampling | How were participants selected? e.g. purposive, convenience, consecutive, snowball | The participants were community health workers (Health Helpers, HH) employed on the *Bukhali* trial; all agreed to participate in the focus groups. HH were recruited using a detailed job description mirroring recruitment criteria as per the South African Department of Health requirements and requiring a high school degree. Additionally, preference was given to candidates between 21-43 years old, and living in Soweto, in an effort to maximise their ability to relate to the participants in the trial. Lastly, preference was given to candidates with some experience in a health-related field. |
| 11. Method of approach | How were participants approached? e.g. face-to-face, telephone, mail, email | Participants were approached through their team leader in a combination of face-to-face and WhatsApp interactions to ask their interest in participating and to negotiate a convenient time to conduct the focus groups. |
| 12. Sample size | How many participants were in the study? | 13 |
| 13. Non-participation | How many people refused to participate or dropped out? Reasons? | 0 |
| *Setting* |  |  |
| 14. Setting of data collection | Where was the data collected? e.g., home, clinic, workplace | At the workplace of the participants |
| 15. Presence of non-participants | Was anyone else present besides the participants and researchers? | No |
| 16. Description of sample | What are the important characteristics of the sample? e.g., demographic data, date | Females between the ages of 23-35 years old, from the community of Soweto, Johannesburg |
| *Data collection* |  |  |
| 17. Interview guide | Were questions, prompts, guides provided by the authors? Was it pilot tested? | The semi-structured interview guide was developed collaboratively by the co-authors; some minor edits to the guide were made after debriefing the first focus group discussion (Supplementary file 3) |
| 18. Repeat interviews | Were repeat interviews carried out? If yes, how many? | No |
| 19. Audio/visual recording | Did the research use audio or visual recording to collect the data? | Audio recorded |
| 20. Field notes | Were ﬁeld notes made during and/or after the interview or focus group? | Field notes were made by the facilitator not asking the questions at certain points of the discussion |
| 21. Duration | What was the duration of the interviews or focus group? | 1 hour 35 minutes, 1 hour 58 minutes, and 3 hours 5 minutes |
| 22. Data saturation | Was data saturation discussed? | Since all participants (community health workers employed at the time of data collection) agreed to participate, the issue of saturation was not directly applicable in this study |
| 23. Transcripts returned | Were transcripts returned to participants for comment and/or correction? | No |
| **Domain 3: analysis and ﬁndings** |  |  |
| *Data analysis* |  |  |
| 24. Number of data coders | How many data coders coded the data? | One author did the initial coding, and this was critically reviewed by one other co-author |
| 25. Description of the coding tree | Did authors provide a description of the coding tree? | The conceptual framework of potential themes and sub-themes was shared with co-authors for their input, before finalizing the coding framework to be applied to the transcripts |
| 26. Derivation of themes | Were themes identiﬁed in advance or derived from the data? | The themes were derived from the data, but they were to some extent influenced by the pre-determined, process-evaluation-driven research questions. |
| 27. Software | What software, if applicable, was used to manage the data? | MAXQDA |
| 28. Participant checking | Did participants provide feedback on the ﬁndings? | The findings were integrated into feedback given in regular debrief sessions with participants as part of their role as community health workers |
| *Reporting* |  |  |
| 29. Quotations presented | Were participant quotations presented to illustrate the themes/ﬁndings? Was each quotation identiﬁed? e.g. participant number | Yes, but only focus group discussion numbers, and not any kind of participant identification, were included, due to the risk of compromising the anonymity of the community health workers |
| 30. Data and ﬁndings consistent | Was there consistency between the data presented and the ﬁndings? | Yes |
| 31. Clarity of major themes | Were major themes clearly presented in the ﬁndings? | Yes |
| 32. Clarity of minor themes | Is there a description of diverse cases or discussion of minor themes? | Yes, where these arose and within the scope of the research question. |
