# Supplementary material for: A qualitative analysis of community health worker perspectives on the implementation of the preconception and pregnancy phases of the Bukhali randomised controlled trial
Source: PLOS Glob Public Health. 2024 Mar 14;4(3):e0002578. doi: 10.1371/journal.pgph.0002578 (PMC10939222; doi:10.1371/journal.pgph.0002578)
Supplement: S2 Text — (PDF) [file pgph.0002578.s003.pdf]

Health Helper focus group guide questions

Introduction
Explain that you are going to cover a range of topics. Firstly you will talk about the intervention content and activities. Then you will ask them about how they identify risk (BMI, BP, Hb etc), refer participants, and help manage the follow up with participants about their risk and referral. Linked to this, you will then talk about how they support participants behaviour change. Lastly, you will also ask them questions about their role as a Health Helper, including the challenges they experience.

Intervention content and activities

- How would you describe the way you go through the content of the intervention materials with participants?
    - What helps you do this well?
        - *Probe:* How this differs for the phases and the way they are designed.
    - What are some of the challenges of going through the intervention content?
    - How do you deal with language barriers, or the participant not fully understanding the information you share with them?
- What is your experience of how participants receive these materials?
    - What topics do they really like / find helpful and/or interesting?
    - What topics do they NOT really like / find helpful and/or interesting?
    - *Probe:* What topics do participants seem the most / least aware about?
- How do participants respond to taking supplements?
    - What is working well with the supplementation?
    - What is NOT working well with the supplementation?
    - How do you help the participate be more aware of the importance of taking supplements?
- How does this differ between phases?

Risk identification, referral and management

- How do you find giving participants feedback about results (key indicators, e.g. BMI, BP, Hb)?
    - Are some results easier / more difficult than others? Are there some that participants don't want to talk about?
    - How do you deal with resistance from participants after receiving results and/or feedback?
    - What challenges do you experience giving this feedback?
    - What helps you to cope with these challenges?
- How do participants receive feedback about results?
    - How well do you think participants understand their results and your feedback?
        - *Probe:* How do participants understand the long-term consequences of these results?
    - How prepared do you feel to help them understand?
        - *Probe:* Do they feel they have the capacity, and the opportunity to do this?
- How do you find using the cue cards?

    - BP, diabetes, iron, BMI, HIV, mental health
- How do you manage participants with an orange or red result?
    - What helps you to manage those with these results?
        - *Probe:* How Healthy Conversation Skills can help with this (especially Open Discovery Questions).
    - What are some of the challenges of managing participants with these results?
- How much time do you spend checking up on referrals?
- How do you find the referral system (i.e. public health system)?
    - What are some of the challenges of the referral system?
        - *Probe:* Referrals for counselling / mental health support.
    - What do you think are some of the barriers participants face in the referral system?
        - What is your role in helping them overcome these barriers?
    - How do participants feel about being referred?
        - What motivates them to attend to take up the referral?
        - How do you influence their motivation to take up the referral?
    - How much do you know about available services for referral?
    - How do these challenges influence the way you manage participants?
- How do you think the services in the trial compare to public health services (HIV and pregnancy testing, ultrasound)?
- How do you think your relationship with participants influences how they use public health services?
- How does this differ between phases?

Supporting behaviour change
- How do you support participants' behaviour change?
    - What are some of the tools you use for supporting behaviour change?
- How do you feel about supporting participants' behaviour change?
    - What influence do you think you have in terms of helping participants to change behaviour?
    - How does this differ between phases (preconception, pregnancy, infancy, early childhood)?
- How do you find using the cue cards?
    - Physical activity, sedentary behaviour, sleep,
- How do participants respond to feedback about their diet?
- What behaviours are easier to change, what are more difficult?
    - What makes them easier / difficult to change?
    - How do you help to overcome these difficulties?
- What makes it easier / difficult for participants to change behaviours?
- How do you find using Healthy Conversation Skills to guide and support this behaviour change?
    - How do you manage goal setting, the SMARTER plan?
    - How do participants respond to these?
- How does this differ between phases?

Your role as a Health Helper

- How would you describe your role as a Health Helper?
  - What does this role require you to BE?
  - What does this role require you to DO?
- For those who have been a Health Helper for a while, how do you think your role has changed over time?
  - What do you think brought about these changes?
- What are some of the expectations of this job?
  - How are you expected to interact with participants?
  - How are you expected to interact with other team members?
  - How are you expected to plan and prioritise your work on a daily basis (especially to meet dosage target)?
  - *Probe:* workload, in terms of participant numbers, phases in the study, fieldwork, process evaluation, capturing data.
- How prepared do you feel for your role?
  - Including previous training and experience, current training and support
  - What other training do you feel you need?
- What are you hoping to achieve in your role?
- What are some of the challenges you experience in your role as a Health Helper?
  - *Probe*: workload, pay, expectations, fieldwork risks (e.g. safety), dealing with participants' trauma.
  - How do you feel when this brings up your own trauma and feelings about your experiences, either current or in the past?
    - How do you cope with this?
  - How do you navigate / manage team dynamics, e.g. working together to achieve a common goal, supporting each other, dealing with a team member who is struggling?
- How do you feel you connect with participants?
  - What does this connection mean to you / how do you feel about this connection?
- How do you feel about providing social support for participants?
  - What are examples of when you have provided social support for participants?
  - What helps you to provide this?
    - *Probe:* their own lived experiences, being able to relate, but also keeping a healthy / professional boundary between your experience and the participant's experience.
  - What makes it challenging to provide this support?
    - *Probe:* How does this take away time from the task at hand, e.g. getting through material for the session?
    - *Probe:* How do you make the participant feel like they are seen and heard, without just focussing on the task at hand?
  - What is your experience of doing things for participants in your own personal capacity?
  - How do you think that sharing your own experiences could make your relationship with a participant (and providing support) challenging (possible negative effects)?
- How do you think your role affected your life (both positively and negatively)?
- What helps you to cope with the challenges you experience in your role?

    - What do you do when you feel this job gets too much?
- What other support do you feel you need?
    - What is the best way to provide this support?
    - Who do you think should provide this support?
- What have been your biggest learnings as a Health Helper?
- What do you understand / know about government CHWs and their roles, responsibilities, and experiences?
    - How does this compare to your experience as a Health Helper?

Closing

- Are there anything else about your role as Health Helpers that you would like to discuss?
