# Supplementary material for: A qualitative analysis of community health worker perspectives on the implementation of the preconception and pregnancy phases of the Bukhali randomised controlled trial
Source: PLOS Glob Public Health. 2024 Mar 14;4(3):e0002578. doi: 10.1371/journal.pgph.0002578 (PMC10939222; doi:10.1371/journal.pgph.0002578)
Supplement: S3 Text — (DOCX) [file pgph.0002578.s004.docx]

Code System

| **Initial conceptual coding framework (prior to generating themes)** |
| --- |
| Implementation: assist with adopting healthy behaviors |
| Obesity and weigh loss |
| HCS/ODQs/SMARTER and participant empowerment |
| Supporting and motivating |
| Other |
| Implementation: provide MMS |
| Family and community |
| Logistical challenges |
| Participant views |
| Other |
| Implementation: risk screening |
| Referrals (challenges) |
| Robot and cue cards |
| Trial vs health services |
| Other |
| Implementation: social support |
| Health literacy gap |
| Language and comprehension |
| Topics, materials, and resources |
| Support delivery |
| Other |
| Additional factors influencing implementation |
| Socioeconomic factors |
| Training |
| Tracing challenges |
