# Supplementary material for: A qualitative analysis of community health worker perspectives on the implementation of the preconception and pregnancy phases of the Bukhali randomised controlled trial
Source: PLOS Glob Public Health. 2024 Mar 14;4(3):e0002578. doi: 10.1371/journal.pgph.0002578 (PMC10939222; doi:10.1371/journal.pgph.0002578)
Supplement: Attachment — Submitted filename: Response Letter_PLOSGPH_25012024.docx [file pgph.0002578.s005.docx]

E-mail: [Larske.soepnel@gmail.com](mailto:Larske.soepnel@gmail.com)

DPHRU, University of the Witwatersrand

South Africa

**Journal:** *PLOS Global Public Health*

15 January 2024

Dear Dr. Hannah Hogan Leslie, editors,

**RE: Response to reviewer comments for manuscript number** PGPH-D-23-02045

**Title:** A qualitative analysis of community health worker perspectives on the implementation of the preconception and pregnancy phases of the Bukhali randomised controlled trial.

Thank you for the useful comments and for the opportunity to revise our manuscript. Please find below a detailed response to each of the reviewer comments, with changes to the text marked in blue (line numbers correspond to the clean manuscript version).

**Comments from editor and journal requirements:**

1. **Please provide separate figure files in .tif or .eps format only and remove any figures embedded in your manuscript file. Please also ensure all files are under our size limit of 10MB. For more information about figure files please see our guidelines:**

[**https://journals.plos.org/globalpublichealth/s/figures**](https://journals.plos.org/globalpublichealth/s/figures) 
[**https://journals.plos.org/globalpublichealth/s/figures#loc-file-requirement**](https://journals.plos.org/globalpublichealth/s/figures#loc-file-requirement)

Thank you, we have ensured that all the figures are in .eps format and do not exceed the size limit.

2. **It would be useful to synthesize the health challenges / desired activities during the preconception period especially, as this period is much less frequently considered than early pregnancy; it would be reasonable to note the same for the pregnant period to the extent it is relevant for this work.**

Thank you for the suggestion, and we agree that the focus on preconception is a novel aspect of the study. We have added this in to the introduction, as follows (see also response to comment 3): (page 3, line 59-78): “The preconception period is increasingly recognised as significant for maternal health outcomes, improved childhood development, and the prevention of NCDs in women and their future children (6,7). This is of great relevance in countries undergoing an epidemiologic transition, such as South Africa, in which escalating NCD risk co-occurs with a high prevalence of infectious diseases (8). For example, in the historically disadvantaged urban setting of Soweto, 66% of women were found to have either obesity or overweight at their first antenatal visit while more than 30% were HIV positive (9). In addition, data from a nutrition screening checklist showed that up to 97.6% of non-pregnant young women reported at least one suboptimal dietary practice (such as not eating enough whole grains or dairy) (10). Preconception care and counselling, including health risk assessment and management, health education, counselling for lifestyle changes such as healthier diets and physical activity, and taking multi-micronutrient supplements (7), are supported by the emerging evidence from interventions in high-income countries (11), but such evidence from South Africa is lacking. Similar to the potential that CHW programs have for health improvement during pregnancy (12), a CHW program could be well placed to overcome some of the challenges of preconception care, such as needing to reach women prior to pregnancy-related healthcare contact and the importance of instilling motivation for lifestyle changes during this period (6). Moreover, among young South African women, CHWs were found to be the preferred agents for delivering a preconception and pregnancy intervention (13).”

3. **Overall, I suggest that the introduction focus more on the health challenges / interventions and implementation context in terms of what is already known from prior work and what is not known - lay the overall basis for existing knowledge on implementation of CHW interventions, preconception interventions, CHW interventions for preconception / pregnancy. The details on Bukhali can be moved to the methods section.**
Thank you for this suggestion, this has been added to the introduction as also described above for comment 2: (page 3, line 59-78): “The preconception period… and pregnancy intervention.”

The focus has also been clarified in terms of the rationale for the aim, as follows: (page 5, line 107-110) “Given the importance of exposures during preconception and pregnancy on future health (16,21,21), more effective implementation of these interventions can contribute to improving the lifetime health trajectories of women in contexts such as Soweto, as well as that of their children. Lastly, it can improve understanding around how CHW roles might be further leveraged to strengthen the healthcare sector outside of the trial, and where potential challenges lie. Therefore, the aim of this paper was…”

The more detailed information on the Bukhali intervention have been moved to the methods (page 6, line 127-133): “Young women living … in the trial.” However, some of the context, rationale, and nature of the trial have been retained in the introduction, as these form part of the justification for the study (for example, the fact that the trial aims to fit into the realities of the south African health and CHW system, and that it is a multi-phase trial).

4. **Recommend use of COREQ or other qualitative checklist to ensure all essential information on data collection, analysis, and interpretation is fully included.**
Thank you, the completed COREQ checklist has been added as Supplementary file 1.

**5. Please describe how HH were initially recruited, since that is essentially the relevant sampling strategy**

This has been added to the “Setting and participants” section (page 6, line 128-133): “HH were recruited using a detailed job description mirroring recruitment criteria as per the South African Department of Health requirements, and requiring a high school degree. Additionally, preference was given to candidates between 21-43 years old, and living in Soweto, in an effort to maximise their ability to relate to the participants in the trial. Lastly, preference was given to candidates with some experience in a health-related field (19).”

**6. Note that results sections are numbered out of order**

Thank you for noting this, section 3.5 has been correctly number as section 3.3.

**7. Presenting the information in the figures as figures adds little value; they could be removed or replaced with a table with more quotes from the discussion groups**.

Thank you for the feedback, we have removed figures 2-7 and have added additional quotes and descriptions to convey (additional) content the figures held.

Reviewer answers to questions:

1. Does this manuscript meet PLOS Global Public Health’s [publication criteria](https://journals.plos.org/globalpublichealth/s/criteria-for-publication)? Is the manuscript technically sound, and do the data support the conclusions? The manuscript must describe methodologically and ethically rigorous research with conclusions that are appropriately drawn based on the data presented.

Reviewer #1: Yes

Reviewer #2: Yes

2. Has the statistical analysis been performed appropriately and rigorously?

Reviewer #1: Yes

Reviewer #2: N/A

3. Have the authors made all data underlying the findings in their manuscript fully available (please refer to the Data Availability Statement at the start of the manuscript PDF file)?

The [PLOS Data policy](https://journals.plos.org/globalpublichealth/s/data-availability) requires authors to make all data underlying the findings described in their manuscript fully available without restriction, with rare exception. The data should be provided as part of the manuscript or its supporting information, or deposited to a public repository. For example, in addition to summary statistics, the data points behind means, medians and variance measures should be available. If there are restrictions on publicly sharing data—e.g. participant privacy or use of data from a third party—those must be specified.

Reviewer #1: Yes

Reviewer #2: Yes

4. Is the manuscript presented in an intelligible fashion and written in standard English?

PLOS Global Public Health does not copyedit accepted manuscripts, so the language in submitted articles must be clear, correct, and unambiguous. Any typographical or grammatical errors should be corrected at revision, so please note any specific errors here.

Reviewer #1: Yes

Reviewer #2: Yes

Comments to the Author

Reviewer #1: Thank to the authors for the study.
1. **Methods: I think it would have helped to hear the voices of the clinic staff responding to some of the allegations around poor services, and patients on why they have less confidence in the formal health services.**

Thank you for your comment. We agree that this would be very useful. Unfortunately, interviews or focus group discussions of clinic staff and participants were outside of the scope of this study, which was a process evaluation of the *Bukhali* trial from the perspective of the health helpers. As mentioned in the discussion (page 22 line 570-571), we hope to explore this further in future analyses of how the trial is interacting with the existing health care system. In addition, we have previously explored the participant perspective in both formative and process evaluation work, and this context has been added to the discussion (page 19, line 507-509): “This lack of management and the anecdotal unpleasant experiences at local clinics has previously been reported by young women in Soweto, including by Bukhali participants (13, 23, 26).”

**2. Under Results, I suggest that at the end of every theme you give a summary as well as a narrative for the figures on how the roles impact on the themes and vice versa.**

Thank you for the suggestion. The figures (2-7) have been removed from the manuscript upon suggestion from the editor. The relevant narrative for the figures not already described has now been integrated into the text of the results (see sections 3.1-3.6), and a summary of the results is given at the beginning of the discussion. In order to avoid too much repetition, we have opted not to add additional summaries at the end of each theme.

**3. Still under results, line 215 has the quote "I was defeated" but the authors did not indicate who made it.**

Thank you for noting this, the relevant quote has been attributed to the correct participant number (page 11, line 254).

Reviewer #2:
**Thanks to the authors for including this qualitative piece to the robust Bukhali trial. Double-checked for “trail” and didn’t find that classic typo – the submission is well-written and clear.**

**Abstract
• Abstract was clear and well written
1. Section header highly recommended.**

Thank you for your comment, the abstract was written without section headers according to the journal guidelines: “do not apply these distinct headings to the Abstract within the article file.”

2**. I feel like the conclusion could be more clear in the abstract, I recommend:**

**Perhaps from line 43 add this from line 503: “These findings provide insights and recommendations for the next phases of the Bukhali intervention, for other CHW-delivered preconception and pregnancy trials, and for the strengthening of ‘504 real-world’ CHW roles in settings with similar implementation challenges.”**

Thank you for the suggestion. We have added this, and have rephrased and shortened this section slightly in order to fit into the 300 word limit for the abstract (page 2, line 39-44).

Introduction:
**3. Clear and well written – I believe the references should include at least one citation from Helen Schneider however, this one comes to mind -** [**https://www.hst.org.za/publications/South%20African%20Health%20Reviews/Chap%207%20WBOTS.pdf**](https://www.hst.org.za/publications/South%20African%20Health%20Reviews/Chap%207%20WBOTS.pdf)

Thank you for this, we have now integrated and listed this reference, as follows: (page 4, line 84-86): “… tensions with facility-based public health care, low wages, and safety concerns and working conditions (2,**16,** 17).”

**4. Aim is clear: “the aim of this paper was to describe factors influencing implementation of the preconception and pregnancy phases of the HeLTI Bukhali intervention, from the perspective of the HHs delivering the intervention.”
Is there anyway to add how this aim might help the health trajectories of women in Soweto overall?**

Thank you for the suggestion. This has been added, as follows: (page 5, line 107-110) “Given the importance of exposures during preconception and pregnancy on future health (6,21,22), more effective implementation of these interventions can contribute to improving the lifetime health trajectories of women in contexts such as Soweto, as well as that of their children.”

Methods
**5 Line 99, I recommend to put ethical considerations at the end of methods.**
This section has been moved to the end of methods section, as suggested.

**6. Ethical considerations, were CHWs compensated for time or transport?
How were FGD facilitators trained? If true could add, “Data collectors were trained in qualitative interviewing techniques, Good Clinical Practice, research ethics and study procedures.”**

The CHWs were compensated for time/transport and refreshments were provided, and this has been added to the “ethical considerations” section (page 8, line 188-189). FGD facilitators (LMS and KM) were trained in qualitative interviewing techniques, research ethics, and study procedures, and the suggested line has been added to reflect this (page 8, line 190-192) (this also reflects in the COREQ checklist, added as supplementary file 1).

**7. Line 135, add “ranging between 1 hour 58 minutes and 3 hours…”**

This has been amended, as suggested.

**8**. **Line 142 data analysis, big concern for me
Please explain why a qualitative data analysis software wasn’t used. Or did I miss it?
o Perhaps excel was used to manage responses/respondents?
o Transcripts in word? Coded in Word?
o And efforts made to protect identity of respondents?**

Thank you for noting this oversight of not mentioning the analysis software. We used MAXQDA analysis software for the data analysis and this has been added to the manuscript (page 8, line 169-170). We have also noted that the participants’ identities were protected through anonymisation of the transcripts prior to analysis (page 7, line 166-167).

**9. Line 146, this is a strength that this is referenced - Analysis was informed by the UKMRC guidance on process evaluations of complex interventions;” However, I strongly recommend adding and reviewing this checklist as part of a robust methods section.**
**o** [**www.equator-network.org/reporting-guidelines/coreq/**](http://www.equator-network.org/reporting-guidelines/coreq/) **♣ One aspect from the checklist that is critical is 32. Clarity of minor themes Is there a description of diverse cases or discussion of minor themes? Similar to CONSORT and STROBE guidance**

Thank you. In addition to the originally submitted SRQR checklist, we have added the COREQ checklist. Point 32 (discussion of minor themes/diverse points) has been expanded upon in the results, as the figures have been removed and any findings not already narratively described, as well as additional quotes, have been added into the text. (See, for example, page 17 line 444-447: “HH expressed that they consider their role as HH, particularly in assisting with healthier behaviours, to have the potential to impact family and community views and behaviour. However, other HH reported that participants would not share health education information, for example about MMS, with their family.”)

**10. Great supplements -** [**https://www.editorialmanager.com/pgph/download.aspx?id=240868&guid=99d98e70-059f-4fae-bbac-d1f5208a6c7c&scheme=1**](https://www.editorialmanager.com/pgph/download.aspx?id=240868&guid=99d98e70-059f-4fae-bbac-d1f5208a6c7c&scheme=1) **Could you add codebook and FGD facilitator guide? (always appreciated by other researchers and good for data transparency in light that the transcripts cannot be shared)**

Thank you for the suggestion, we have added the FGD topic guide and the codebook as supplementary files 2 and 3.

**Results
11. Recommend at the beginning of the results the authors explain the outline of the results and themes – I think this is the intention of the Figure. This should be briefly mentioned in text.**

Thank you, this has been added to the text, as follows: (page 9, line 195-202) “In this section, each of the following six themes will be presented as factors influencing implementation: (i) interaction with existing public healthcare sector; (ii) participant perceptions of health; (iii) health literacy and language barriers; (iv) participants’ socioeconomic constraints; (v) family, partner, and community views of trial components; and (vi) the HH-participant relationship. Per theme, the relevant tools used by the HH to assist with implementation will also be described. Figure 1 provides a conceptual overview of how the themes impact the implementation of the four HH roles, and, conversely, how enactment of these HH roles can impact the identified themes.”

**12. Great selection of quotes! “They won’t go straight to the clinic, so they will always start with us first to find out what we know, what we think, before they go to the actual clinics.” (FGD 2)
• Line 242: I recommend the assessment of “flawed methods such as scaring participants into action” into the discussion. As it’s not purely a data point, but this is a tricky decision.
♣ My assessment of the included quotes is that the HHs aren’t trying to scare. But maybe I’m misunderstanding.
♣ An alternative might be to include this in the Discussion and include elements from the Health Belief Model – “perceived severity” as a motivation.
♣** [**https://sphweb.bumc.bu.edu/otlt/MPH-Modules/SB/BehavioralChangeTheories/BehavioralChangeTheories2.html**](https://sphweb.bumc.bu.edu/otlt/MPH-Modules/SB/BehavioralChangeTheories/BehavioralChangeTheories2.html) **♣** [**https://www.magonlinelibrary.com/doi/full/10.12968/ajmw.2021.0012#:~:text=For%20example%2C%20the%20health%20belief,barriers%20and%20exposure%20towards%20cues**](https://www.magonlinelibrary.com/doi/full/10.12968/ajmw.2021.0012#:~:text=For%20example%2C%20the%20health%20belief,barriers%20and%20exposure%20towards%20cues)

**♣ Does Figure 3 suggest “scaring” is recommended tool?**

Thank you for your comment. We agree that the selected quotes did not reflect this conclusion very clearly, and we have added an additional quote which indicates the self-described use of this strategy by the HH: (page 12, line 297-300) ““We basically need to show them the dangers of it. Even if it means us scaring them … The moment you tell them that “you will be dying in the next two months” that’s when they become scared. So somewhere, somehow, I think we need to put in a little bit of danger so that they can see how serious this is.” (FGD 3)”.

The link between this strategy and the “perceived severity” aspect of the Health Belief Model is very interesting, and we have included to this in the discussion (page 20, line 539-543): ‘…adverse tactics such as using exaggerated claims to ‘[scare] participants into action’. Such training could include, for example, bio-ethical considerations around fear appeals, and their potential, in the framework of the health belief model, for influencing behaviour change through increased perceived severity and susceptibility, only if used appropriately (43–46).”

Figure 3 was not intended to explicitly list this strategy as a recommended tool, but merely as one that was reported by HH as being used. However, we agree that this was presented somewhat ambiguously in the figure, which has been removed (upon recommendation from the editor that these figures do not contribute significantly to the manuscript).

**13. Line 321, should it be HH here?**

Thank you, this description has been removed based on reviewer feedback.

**14. Line 330 – “preferable.”**

This has been edited as suggested.

**15. Line 331 – maybe I’m purest, but I would exclude the 11 languages of South African in the results – unless there’s a quote that captures it.**
Thank you, this has been removed.

**Discussion
16. Very helpful to have sub-headers like the results. I recommend (at least):
o Alignment to other literature
♣ This is well covered around line 460
o Application to Bukhali adaptations
o Strengths and limitations
o Recommendations (repeat of adaptations?)
♣ Line 450 an existing example, could these all be in one place?**

Thank you for the suggestion; we have added sub-headers in the discussion to clarify the structure of the discussion, in which the three “broadly applicable strategic areas” are discussed in terms of alignment with the existing literature, recommendations for *Bukhali*, and for other HH programmes, followed by the “strengths and limitations” section (sections 4.1 Navigating and bridging the health care system-4.3 Strengths and limitations). We have additionally clarified this structure by adding the following sentence to the end of the first paragraph of the discussion (page 18, line 495-496): “These strategies and how they align with existing literature are discussed in more detail below.”

**17. I think the Strengths and Limitations section could be enhanced.
o The mention of power dynamics was a great addition. I recommend considering these classics:
o Limitations – inability to generalise to a large population, researcher bias – all mitigated by the important strengths of these results to improve Bukhali as well as larger programmes to support this population
o Strengths –
♣ embedded into a larger multi-year trial
♣ Minimal risks to participants in the trial
♣ HHS provide a critical viewpoint, able to speak for themselves as well as the women the trial is studying
o From the COREQ mention in methods, this could provide some** **guidance** [**https://academic.oup.com/intqhc/article/19/6/349/1791966**](https://academic.oup.com/intqhc/article/19/6/349/1791966)

Thank you. The strengths and limitation section has been enhanced, as suggested: (page 21, line 565-568) “One strength of the study is that it was part of a large, multi-year trial, and that it offered a unique viewpoint and provided a voice for the HHs, who work more closely with the trial participants than any other trial staff and therefore provide valuable insights. The study also presented minimal risks to the participants of the trial or to the HHs. Additionally …” and (page 22, line 579-582) “Lastly, the qualitative results may not be applicable to other diverse trial populations, and (as is inherent to reflexive analysis methods), analyses is influenced by researcher subjectivity. Nevertheless, the results can provide deeper insights for Bukhali as well as other HH programmes.”
